# News from Societies

**Published:** 1957-10

**Authors:** 


### News from Societies

### PROGRAMMES 1957–58 SESSION

### BRISTOL MEDICO-CHIRURGICAL SOCIETY

9th Oct.: **Annual General Meeting and Presidential Address.**

13th Nov.: **Clinical Meeting** at Bristol Royal Infirmary.

11th Dec.: Mr. R. H. Belsey, Dr. J. E. G. Pearson *et al.* **"Lung Cancer".**

8th Jan.: Professor C. H. Stuart-Harris, **"Diseases in search of Viruses".**

12th Feb.: Professor J. M. Yoffey, **"The Lymphatic System"** (Film and Commentary).

12th Mar.: Mr. S. D. Loxton, **"Common Obstetric Emergencies".**

9th Apr.: Dr. C. T. Andrews, **"The Story of John Keats, Apothecary and Poet".**

14th May: Dr. W. A. Gillespie, **"The Hospital Staph.".**

### THE DEVON AND EXETER MEDICO-CHIRURGICAL SOCIETY

3rd Oct., at 8.15 p.m.: **Meeting with Clergy** in Bishop's Palace.

17th Oct.: **Clinical Meeting.**

3 p.m. Cases on view in Medical O.P. Department.

4 p.m. Tea in Library followed by Discussion of Cases.

7th Nov., at 4 p.m. Joint Meeting with B.M.A. F. E. Camps, Esq., M.D. (Reader in Forensic Medicine, University of London), **"Truth is stranger than Fiction".**

(Exeter South-Western Dinner).

21st Nov., at 8.15 p.m.: **Clinico-Pathological Demonstration.**

12th Dec., at 8.15 p.m.: **Clinical Meeting** at the Princess Elizabeth Orthopaedic Hospital.

16th Jan., at 8.15 p.m.: Dr. Herbert Constable (Deputy Secretary of the Medical Protection Society), **"Medico-legal Hazards of the Doctor and Surgeon".**

6th Feb., at 8.15 p.m.: T. N. Fison, M.D., **"Two Cases of Glomerular-nephritis in Infancy"**, and L. N. Jackson, M.C., M.D., **"A Notable case of Obesity".**

27th Feb.: **Clinical Meeting.**

3 p.m. Cases on view in Medical O.P. Dept.

4 p.m. Tea in Library followed by Discussion of Cases.

20th Mar., at 8.15 p.m., Professor J. McMichael, F.R.S., M.D., F.R.C.P., The Post Graduate Medical School, **"Hypertension".**

17th Apr., at 8.15 p.m.: **Annual General Meeting** and **Clinico-Pathological Demonstration.**

### WEST OF ENGLAND CHILD HEALTH GROUP

24th Oct., at 5.30 p.m.: Miss L. A. E. Shaw, M.A., Lecturer in Social Studies, University of Bristol; and Miss M. E. Davies, Health Visitor Tutor, Welsh National School of Medicine, Cardiff. **"Discussion on the Health Visiting Report".** Department of Child Health Lecture Theatre, Children's Hospital, Bristol.

28th Nov., at 8.30 p.m.: Miss C. N. Grose, S.R.N., S.C.M., Assistant Matron, British Hospital for Mothers and Babies, Woolwich. **"Breast Feeding".** Department of Child Health Lecture Theatre, Children's Hospital, Bristol.

12th Dec., at 5.30 p.m.: K. Laybourn, Esq., PH.D., M.SC., Chief Inspector of Schools, Bristol. **"Selection of Children for Secondary Education".** Department of Child Health Lecture Theatre, Children's Hospital, Bristol.

16th Jan., at 8.30 p.m.: Kent Paediatric Society. Department of Child Health Lecture Theatre, Children's Hospital, Bristol.

6th Feb., at 8.30 p.m.: Miss J. M. Hood, M.A., Colonial Liaison Officer, Committee for the Welfare of Colonial Workers in Bristol. **"West Indian Families".** Department of Child Health Lecture Theatre, Children's Hospital, Bristol.

20th Mar., at 6.30 p.m.: By invitation of J. Macrae, Esq., M.D., D.P.H., Consultant Physician. **Clinical Meeting.** Ham Green Hospital, Pill, Nr. Bristol.

May. (Date to be announced later). W. C. W. Nixon, Esq., F.R.C.S., M.D., Director, Obstetric Unit, University College Hospital. **"Obstetrics from the Preventive Medicine Aspect".** Department of Child Health Lecture Theatre, Children's Hospital, Bristol.

June. (Date to be announced later). F. R. G. Heaf, Esq., M.A., M.D., F.R.C.P., David Davies, Professor of Tuberculosis, University of Wales. **Field Visit and Talk.** Department of Tuberculosis, University of Wales, Cardiff.

### B.M.A. (Cornwall Division)

28th Nov.: **Dinner and Dance.** Hotel Bristol, Newquay.

December. A Medical Film Show in co-operation with the West Penwith Medical Society.

11th Apr.: B.M.A. Clinical Lecture by H. Osmond-Clarke Esq., C.B.E., F.R.C.P., on **"Pain in the Arm and Neck".**

### B.M.A. (Exeter Division)

7th Nov., at 3.45 p.m.: Joint Meeting with Devon and Exeter Medico-Chirurgical Society. F. E. Camps, Esq., M.D., **"Truth is stranger than Fiction".**

### B.M.A. (Gloucestershire Branch)

10th Oct., at Cheltenham: Presidential Address. Mr. R. Harvey, **"Doctor in Prison".**

14th Nov., at Gloucester: **Clinical Meeting.**

12th Dec., at Cheltenham: Dr. Mandelbrote, **"Psychiatric Problems in General Practice".**

9th Jan. at Gloucester: Film by Dr. G. R. Fearnley, followed by a Paper by Dr. Hope-Simpson, **"Research in General Practice".**

13th Feb., at Cheltenham: Dr. T. A. Pitt-Evans and Dr. Niven. **Demonstration of Laboratory Techniques and other clinical applications.**

13th Mar., at Gloucester: Dr. A. Tom. (Members of the Dental profession to be invited to attend.) Paper on **"Nitrous Oxide Anaesthesia"** and a film or demonstration of cases.

10th Apr., at Cheltenham: **B.M.A. Lecture.**

8th May, at Lydney: **Annual General Meeting.**

12th June, at Cheltenham: **President's Guest.**

The Cheltenham Meetings will be held at the Cheltenham General Hospital; the Gloucester Meetings will be held at Good Templar Hall, Park Road, Gloucester, with the exception of the Clinical Meeting in November, which will be held at Gloucester Royal Infirmary.

### B.M.A. (South-Western Branch)

28th Nov., at 3 p.m : **Clinical Meeting.** Torbay Hospital, Torquay.

### B.M.A. (West Somerset Division)

25th Oct.: **Dinner** with British Dental Association, at the County Hotel, Taunton. Mr. Horsnell, of the London Hospital Dental School, **"Diet".**

8th Nov.: **Joint Dinner** with the West Country Law Society at the County Hotel, Taunton. Mr. Skelhorn, **"Legal responsibilities of Wives".**

16th Nov., at 2.30 p.m.: Clinical Meeting at Musgrove Park Hospital, Taunton, **"Disorders of the Locomotor System".**

28th Nov.: **Sixth Annual Dinner and Dance** of the West Somerset Division at the County Hotel, Taunton.

Late February, 1958: Lecture: **"Recent Advances in the Treatment of Cancer".** Yeovil Hospital, Yeovil.

### BRISTOL SOUTH MEDICAL GROUP

Meetings take place on the first Sunday in each month during the winter at the Bristol General Hospital at 8 p.m.

### COSSHAM MEDICAL SOCIETY

8th Oct.: J. M. Cormack, M.B., CH.B., D.P.H., **"Presidential Address".**

5th Nov.: J. D. Kershaw, M.D., B.S., D.P.H. (late Chief United Nations Rehabilitation Unit), **"The Welfare of the Handicapped Child".**

3rd Dec.: W. H. Bradley, D.M., M.R.C.P., J.P., Ministry of Health (Senior M.O.), **"Control of Infectious Diseases".**

7th Jan.: G. D. Roberts Esq., Q.C., Recorder of Bristol, **"Experiences of Forsenic Medicine".**

4th Feb.: To be announced later.

4th Mar.: E. C. Morris Jones, M.B., B.S., B.H., D.P.H., D. M. Waddington, M.B., CH.B., D.P.H., Dr. Sarah Walker, M.D., D.P.H., **"Co-ordination in Maternity and Child Welfare".**

18th Mar.; **Buffet Dance,** Ashton Court Country Club.

1st Apr.: **Annual General Meeting.**

Meetings held at Cossham Hospital. Coffee at 8.45 for 9 p.m. Meeting.

### BATH CLINICAL SOCIETY

All Meetings will be held at The Royal United Hospital, Bath, except that on 10th Jan., which will be at St. Martin's Hospital, Bath.

11th Oct., at 8 p.m.: **Clinical Meeting.**

8th Nov., at 8 p.m.: Cases, followed by A. Daunt Bateman, Esq., O.B.E., F.R.C.S., on **"Deafness in Children".**

13th Dec., at 8.30 p.m.: Ashton Miller, Esq., F.R.C.S., on **"Micturition Disorders in Child and Adult".**

10th Jan., at 8.30 p.m. at St. Martin's Hospital, Bath. Joint Meeting with The Bath Law Society. (To be decided.)

14th Feb., at 8.30 p.m.: Short papers, preceded at 8 p.m. by illustrative cases.

14th Mar., at 8 p.m.: Cases, followed by Dr. Lycett, D.P.H., on **"Prophylactic Inoculation and Vaccination: the present position".**

4th Apr., at 8.30 p.m.: T. F. Coade Esq., M.A., Headmaster of Bryanston School, on **"Adolescence".**

3rd May: **Annual Dinner** at Fortt's Restaurant, Bath.

### CORNWALL CLINICAL SOCIETY

*Secretaries:* Dr. J. D. Hardy and Dr. B. A. Gwynne Jenkins.

4th Oct.: **Clinical Evening,** Redruth Hospital.

7th Nov.: Films: **"William Harvey and the Circulation of the Blood"** and other Films. Royal Cornwall Infirmary.

28th Nov.: **B.M.A. Dinner and Dance.** Hotel Bristol.

6th Dec.: Dr. Fox, Editor, *Lancet.* **"Medicine in Soviet Russia"** (Illustrated). Royal Cornwall Infirmary. (Ladies' Night.)

10th Jan.: **"Any Questions".** A Medical Quiz with Panel of Experts. Redruth Hospital.

31st Jan.: N. S. Barrett, M.S., F.R.C.S., **"The Evolution of Cardiac Surgery and its Present Scope".** Royal Cornwall Infirmary.

6th Mar.: **Clinical Evening.** Falmouth Hospital.

11th Apr.: H. Osmond-Clarke, C.B.E. B.M.A. Lecture, **"Pain in the Arm and Neck".** Royal Cornwall Infirmary.

1st May: **Clinical Evening.** Royal Cornwall Infirmary.

6th June: Cases and Demonstrations by Consultant Staff. Redruth Hospital.

4th July: **Annual General Meeting and President's Address.** Redruth Hospital.

### WESSEX RAHERE CLUB

26th Oct., at 7.30 for 8 p.m.: **Autumn Dinner** at Lansdown Grove Hotel, Bath. Dr. R. I. W. Ballantine of Bart's, will be Guest of Honour.

### SOCIETY OF MEDICAL OFFICERS OF HEALTH

### WEST OF ENGLAND BRANCH

5th Oct.: Dr. J. Reynolds, M.C. **Presidential Address.** Taunton.

4th Jan.: Bristol.

5th Apr.: Bristol or Bath.

May: Joint Meeting with Southern Area of the Branch at Palace Hotel, Torquay.

### TORQUAY AND DISTRICT MEDICAL SOCIETY

31st Oct., at 4.0 p.m. Opening Meeting. Miss Josephine Barnes, D.M., M.R.C.P., F.R.C.S., F.R.C.O.G., **"Current Problems in Obstetrics".**

7.45 for 8.15 p.m.: **Dinner and Dance** at the Palace Hotel, Torquay.

19th Nov., at 8.30 p.m.: Arranged by the Fellowship of Doctors and Clergy—Dr. Robert Mortimer, Lord Bishop of Exeter, **"The Care of the Aged".**

28th Nov., at 3 p.m.: **Combined Clinical Meeting** with S.W. Branch of British Medical Association.

12th Dec. at 8.30 p.m.: Dr. D. Stafford-Clark, F.R.C.P., **"The Nature of Psychopathic Personality".**

23rd Jan., at 8.30 p.m.: Dr. R. H. Mole, A.E.R.E., Harwell, **"Atomic Energy and Its Hazards".**

13th Feb., at 8.30 p.m.: Victoria Hotel, Torquay, **Clinical Discussion.** Opened by Dr. M. G. Thorne.

13th Mar., at 8.30 p.m.: Dr. Richard Asher, F.R.C.P., **"Face Values".**

24th Apr., at 8.30 p.m.: Arthur Dickson Wright, Esq., M.S., F.R.C.S., **"Some Historical Operations".**

29th May, at 3 p.m.: Hawkmoor Chest Hospital. Annual General Meeting and Summer Meeting. **"Demonstration of Lung Function Tests in relation to Emphysema, Bronchitis and Asthma".**

### WEST PENWITH MEDICAL SOCIETY

Sept. 19th: Dr. A. S. Thorley, of Belmont Hospital, Sutton. Films: **"Hypoglycaemic coma in Psychiatry and other subjects".**

Oct. 17th: **Annual Dinner.**

Nov. 21st: **"The Open Laboratory".** West Cornwall Hospital.

19th Dec.: Film. Presented by the British Medical Association.

16th Jan.: H. V. Wingfield, Esq., F.R.C.S., **Symposium on Lung Cancer.**

20th Feb.: **Clinical Meeting.** Members of the Hospital Staff.

20th Mar.: Dr. Mark Hewitt. **"Some Skin Diseases".**

17th Apr.: K. Rostron, Esq., F.R.C.S. **Symposium on Headaches.**

15th May: Dr. Anne Gibson of the South London Hospital for Women. **"A Background to Bacteriology".**

19th June: To be arranged.

17th July: On a Medico-Legal Subject.

Meetings are held at the West Cornwall Hospital, Penzance.

